# Book Review

**Published:** 1922-06

**Authors:** 


### Reviews of Books.

**Clinical Surgical Diagnosis.** By F. DE QUERVAIN. Third English Edition. Translated from the seventh edition by J. SNOWMAN, M.D. Pp. xvi., 914. London: John Bale, Sons and Danielsson, Ltd. 1921. Price £2 10s. net.—This work is written in a fresh and conversational manner, and in places one can almost imagine that the text had been taken down *verbatim* as Professor de Quervain stood by a bedside and demonstrated a case to a class of students. The author assumes throughout that his readers have mastered the elementary principles of routine examination, and are capable of recognising the simpler surgical pathological conditions. It will prove more valuable, therefore, to the senior student and the post-graduate than to the beginner. The two outstanding features of the book are, firstly, the great stress which is laid upon the value of the clinical history in effecting a diagnosis, and secondly the illustrations, which call for unqualified praise. Their acquisition must have entailed long and painstaking labour. As is natural in a book by a single author, which covers such a large field, it is somewhat unequally written, but we would especially commend that portion which deals with the diagnosis of abdominal conditions. Undoubtedly the book will prove a very valuable aid in the solution of many a puzzling diagnostic problem.

**The Early Diagnosis of Tubercle.** By CLIVE RIVIERE, M.D., F.R.C.P. Third Edition. Pp. xvi., 318. London: Oxford Medical Publications. 1921. Price 15s. net.—Two years have elapsed since the appearance of the last edition, the chief addition being to the section of Differential Diagnosis, with an account of pulmonary syphilis and pneumo-mycosis. The reputation of the book as an authoritative and masterly presentation of one of the most difficult problems in medicine is well established. Criticism can be directed only to the amount of the information. We feel the work would receive a greater recognition if the author would quote more from his own experience and give less prominence to the work of others. The chapter on the value of X-rays in diagnosis gains in interest in that Dr. Riviere states definitely his opinion of the interpretation of such findings. He also holds out the hope that in future editions he will prune that portion dealing with specific diagnosis. As he himself says, "the amount of space devoted to tuberculin . . . . will be found to correspond more closely with the claims . . . made for it, than with its actual worth in diagnosis." The scheme of physical examination outlined by the author requires endurance of high degree both on the part of the patient and certainly of the physician.

**The Clinical Study of the Early Symptoms and Treatment of Circulatory Disease in General Practice.** By R. M. Wilson, M.B. Pp. xvi., 245. London: Oxford Medical Publications. 1921. 15s. net.—In this little book Dr. Wilson enlarges upon matters already considered by him in the other books he has published: the mechanism by which the speed of the heart is altered in disease, the origin of dyspnœa, and so forth. The method which he has adopted—the observation and correlation of clinical phenomena—is worthy of all encouragement. Not medicine alone, but also human physiology and pathology, owe more to such observations than to laboratory work, divorced from the bedside as it so often is. In the adoption of the clinical method Dr. Wilson is indeed a follower of Sir James Mackenzie, to whom the book is dedicated. And there is another aspect of that distinguished exemplar's work which may best be summed up in the opening sentence of his preface to the first edition of the *Diseases of the Heart,* "In the following pages are given the results of observations on affections of the heart, made during an active practice of over a quarter of a century." Dr. Wilson might do worse than let his ideas ripen and his observations accumulate, for as long a period, before committing them to print.

**The Clinical Examination of the Nervous System.** By G. H. Monrad-Krohn, M.D. Pp. xv., 135. London: H. K. Lewis & Co. Ltd. 1921. Price 6s. net.—This book gives a very full and clear account of the systematic method of examination of the nervous system, including an outline of the examination of the mental state of patients. Appendices also deal with Binet-Simon tests, diplopia and vestibular tests. There are also several very useful anatomical diagrams. It should prove most helpful to students and house physicians.

**On Modern Methods of Treating Fractures.** By Ernest W. Hey Groves, M.S., M.D., F.R.C.S. Second Edition. Pp. xix., 435. Bristol: John Wright & Sons Ltd. 1921. Price 30s. net.—The new edition of this excellent book well repays study. It has been largely re-written, and the majority of the illustrations are new. The author embodies in it his extensive experience obtained during the war, and introduces all good modern methods of the treatment of fracture in addition to his own. He emphasises the fact that each has its special application, and that a combination of two or all is always essential to the most satisfactory results, each performing a special function and being founded on some definite principle which is complementary to the other methods. The Report of the Fracture Committee of the British Medical Association showed that the function obtained varied largely with the excellence or otherwise of anatomical alignment following fracture. The methods of attaining good alignment are considered under three headings. *Massage and Movements*, which need be the only treatment where no serious displacement has occurred. *Extension*, which should be applied where operation is refused or unsuitable and for fractures in children where it is most successful. *Operation* for gross deformities, especially in the leg bones, for fractures in the adult and when a week's extension and massage have failed to bring about reduction. From the author's experiments, which have been largely devoted to operative methods of accurate alignment and fixation, the fact emerges that these ends have been best attained by means of plates fixed by long bolts or pins or by strong intramedullary pegs. The history and literature of bone grafting is more fully reviewed and the author's experimental work on this is given in detail together with instances of its practical application in the treatment of fractures.

**A Physical Interpretation of Shock, Exhaustion, and Restoration.** By George W. Crile, M.D. Pp. xvi., 232. London: Oxford Medical Publications. 1921. Price 25s. net.—This large and well illustrated volume gives a full exposition of the author's extensive and far-reaching studies on the nature of surgical shock. The metabolic changes, the alterations in the nerve-cells and those of the liver and other organs, the distribution of the blood in the vascular system, and the relation of shock to the secretions of the endocrine glands are explained, and some sort of a theory of shock evolved. There is a good chapter on prevention and treatment, and if this follows what are now well-known lines it should be borne in mind that Crile's previous publications have contributed not a little to the settlement of those lines. He is more earnest than some observers in his advocacy of opium as a remedy for shock. Unfortunately the bibliography, full as it is, was evidently collected before the publication of the important British researches at the conclusion of the war, and therefore the book cannot be described as quite up to date. Also the author's speculations as to the exact nature of the intra-cellular changes that take place in shock will need a good deal of physiological examination before they can be considered as of much importance.

**The Journal of Metabolic Research.** Edited by FREDERICK ALLEN. Published by the Physiatric Institute, Morristown New Jersey. Price $10.00 per year.—We have received for exchange the first number of a new journal which is intended to serve for the publication of the results of original research in the problems of metabolism. The Editor, Dr. F. M. Allen, whose work on Diabetes is widely appreciated, explains how the *Journal* has come into existence. A series of papers on diabetic pathology found such difficulty of acceptance by other journals on the score of length and expense, that after ascertaining that this difficulty had been experienced by other workers in the same field, it was decided to establish this *Journal of Metabolic Research.* The Editor hopes to be able to publish papers promptly and at full length, stipulating only that they shall contain significant facts in the briefest form compatible with completeness. The expense of such a publication must necessarily be high, and apparently the Institute of Physiatric Research is undertaking the financial responsibility involved. The first issue contains a number of papers by Allen and his fellow-workers dealing in the main with observations upon experimental diabetes. They are of the greatest interest, and leave us in no doubt that the *Journal* will rank amongst the most valuable of the periodicals devoted to experimental pathology.

**Intrinsic Cancer of the Larynx and the Operation of Laryngo-fissure.** By IRWIN MOORE, M.B., C.M. Pp. xii., 147. University of London Press. 1921. Price 20s. net.—The very high percentage of permanent success now obtainable from the operative removal of intrinsic cancer of the larynx, provided the nature of the growth be recognised while still in a favourable position, renders this authoritative and detailed exposition of the subject most opportune. The instruments devised by the author are widely known and used by laryngologists, and are the outcome of his exceptional opportunities for improving the technique of laryngo-fissure for laryngeal cancer. Success in these operations depends greatly on minute details, firstly in the diagnosis, then in the preparation of the patient, on the operation, but lastly and not least on after treatment. All these aspects of the question are fully considered, and the author is not content to relate his own methods, but throughout affords reasoned argument with abundant citation of the work of other authorities, in support of his advocacy of methods which have such important bearing on results. The book is profusely illustrated, most of the drawings being both original and very helpful. The author is to be congratulated on the work comprised in this small volume.

**Bowel Diseases in the Tropics.** By Sir LEONARD ROGERS, C.I.E., M.D. Pp. xvi., 475. London: Oxford Medical Publications. 1921. Price 30s. net.—Sir Leonard Rogers has combined his works *Cholera and its Treatment* (1911) with *The Dysenteries, their Differentiation and Treatment* (1913) under the above title; an additional final short chapter on Hill Diarrhœa and Sprue completes the volume. The hypertonic saline treatment in cholera and the emetine treatment of amœbiasis, two subjects to which the distinguished author has made such notable and original contributions in Therapy, are treated in the greatest detail. Table XXI., confirmatory of the value of the hypertonic saline and permanganate treatment, is very instructive. The mortality of the writer's cases is 22.35 per cent. (2,009 treated), as compared with 59 per cent. (1,243 treated) in previous years. On page 288 an analysis of 32 consecutive cases of amœbic dysentery treated by emetine illustrates the very considerable lowering of mortality, 10 per cent. as against 43.3 per cent. in the ipecacuanha series, and the still more satisfactory shortening of the period of dysenteric symptoms to 2.35 days from 11.4 in the other series. In discussing the late complication of cholera, namely suppression of urine, wrongly called, we think, uræmia, Sir Leonard quotes the blood pressure findings in such cases. It is quite clear from the text that the suppression of urine is intimately connected with the circulatory failure, pressures below 100 mm. Hg being associated with anuria. There are six beautiful coloured plates illustrative of the dysenteric processes both amœbic and bacillary. One is perhaps unique, the result of one week's treatment with emetine, death from perforation of the cæcum. The mucous membrane is destroyed extensively in several places as far down as the peritoneal coat. As the author points out, such a condition is not compatible with recovery. Diagram 3, based on an analysis of over 100 *post-mortem* examinations of dysentery, emphasises in a graphic manner the distribution of the lesions in the various clinical types of dysentery, both acute and chronic. The book should, we feel, be received with gratitude by all whose work lies in the tropics. It is packed with information, statistical, clinical, and therapeutic; the recent work in the etiology of the dysenteries receives full recognition. We hope that future editions will consider sprue in greater detail.

